# The scientific, the literary and the popular: Commerce and the reimagining of the scientific journal in Britain, 1813–1825

**DOI:** 10.1098/rsnr.2016.0027

**Published:** 2016-09-21

**Authors:** Jonathan R. Topham

**Affiliations:** School of Philosophy, Religion and History of Science, University of Leeds, Leeds LS2 9JT, UK

**Keywords:** scientific journals, popular science, science and literature, scientific publishing, late Georgian Britain

## Abstract

As scientists question the recent dominance of the scientific journal, the varied richness of its past offers useful materials for reflection. This paper examines four innovative journals founded and run by leading publishers and men of science in the 1810s and 1820s, which contributed to a significant reimagining of the form. Relying on a new distinction between the ‘literary’ and the ‘scientific’ to define their market, those who produced the journals intended to maximize their readership and profits by making them to some extent ‘popular’. While these attempts ended in commercial failure, not least because of the rapidly diversifying periodical market in which they operated, their history makes clear the important role that commerce has played both in defining the purposes and audiences of scientific journals and in the conceptualization of the scientific project. It also informs the ongoing debate concerning how the multiple audiences for science can be addressed in ways that are commercially and practically viable.

*Philosophical Transactions* established the four fundamental principles (registration, verification, dissemination and archiving) still in use by the almost 30 000 science journals today. But science publishing has remained almost unchanged until a few decades ago and the introduction of the internet.^1^

The advent of digital media has prompted scientists and historians of science alike to re-examine communication processes that had hitherto seemed to be essential features of the scientific project. For much of the twentieth century, the peer-reviewed scientific journal article was so firmly entrenched as the touchstone of scientific knowledge that alternatives seemed unthinkable. Moreover, teleological histories—like the one quoted above—suggested that this defining characteristic of science had been a fixture since the Scientific Revolution of the seventeenth century, beginning with the *Philosophical Transactions* of the Royal Society in 1665. Faced, however, with a rapidly changing communication environment following the advent of the Internet, and with questions arising concerning the effectiveness of peer review, the value of journal impact factors and the practicality and propriety of having leading journals marketed for princely sums, scientists have found themselves questioning the fitness for purpose of the scientific journal article. At the same time, historians have been increasingly eager to expose the inadequacy of allowing the twentieth century's obsession with the format to dictate the terms in which we understand the past. The scientific journal did not appear in anything like its modern form in the seventeenth century. Rather, the periodical scientific publications produced over the last third of a millennium have been profoundly variable, undergoing repeated reimagining and experimentation, as the projects and communities of natural enquiry and the marketplace and technologies of print communication have changed. In these new histories, it transpires that many of the defining features that we associate with the scientific journal—above all, peer review as the authenticating token of science—are actually very recent.^2^

For those engaged in the contemporary debate about the future of the scientific journal, such historical insights surely have much to offer. At a moment when fundamental questions are being asked concerning the nature and purposes of scientific communication, the proper audiences for such communication and the involvement of commercial interests in the process, there is much to be gained by rediscovering how these have changed over time and examining the issues and possibilities that arose in the past. The purpose of this paper, therefore, is to explore one particular historical moment—which occurred some 200 years ago—when serious and considered attempts were made in Britain to reimagine what were then called scientific journals. That moment occurred in the immediate aftermath of the Napoleonic Wars, at a period when the sciences were rapidly being reorganized, when technologies of communication were undergoing radical change and when reading audiences were expanding and diversifying out of all recognition. In the midst of these dramatic changes, a small number of scientifically educated editors and leading fashionable publishers decided the time was ripe for scientific journals to expand and develop to meet their potential both in improved contents and in sales and public reputation. As today, the core question was what a scientific journal should be and do. Who should write and read about science? In what form? With what purposes? And how should it be made to pay? The answers to these questions were strikingly different from the answers we are accustomed to hearing, but the discussion and the outcomes raised enduring issues of lasting interest.

The paper is divided into two main sections. It begins by situating the innovative journals of the 1810s and 1820s within the longer history of scientific journals in Britain, establishing that the first publications designated as ‘scientific journals’ were commercial speculations produced in the rapidly growing book trade of the late eighteenth century in contrast to the transactions of learned societies. As this market developed, publishers and editors developed important distinctions—between the ‘literary’ and the ‘scientific’ and between the ‘popular’ and the ‘technical’—to demarcate the intended audience for their new products. In particular, they sought to maximize their readership by making their scientific contents at least partly ‘popular’. The second section explores in more detail this process of reimagining the scientific journal in which the editors and publishers of the 1810s and 1820s engaged, outlining their concerns about how to enlarge the readership of their publications, not least through a ‘popular’ mode of address, and the difficulties that they experienced in doing so. The new journals ultimately had little success, and the section ends by showing that this was at least in part because of the manner in which the periodical market diversified, addressing different groups of readers separately. Finally, I draw out the wider historiographical importance of this account in refocusing historical attention on the commercial nature of much scientific communication and its consequences for conceptualization of the scientific project, and consider the value of the history in rethinking the future of scientific journals.

## Situating ‘scientific journals’ in early nineteenth-century Britain

Although the earliest periodical scientific publication in Britain—the *Philosophical Transactions* of the Royal Society—commenced in 1665, the term ‘scientific journal’ (or, more commonly at first, ‘philosophical journal’) did not come into use in English until the end of the eighteenth century. When it did, it signalled a contrast between the new commercial journals produced by entrepreneurial booksellers, and the existing luxurious transactions of learned bodies such as the Royal Society. The new commercial ‘philosophical journals’ were the latest facet of an expanding magazine market that was a defining characteristic of eighteenth-century Britain. Beginning with the *Gentleman's Magazine* in 1731, the new ‘magazines’ were designated in a way that identified them as constantly growing ‘storehouses’ of knowledge on all subjects, including ‘philosophical’ topics. In this way, they sought to fulfil the encyclopaedic ambitions of Enlightenment culture, offering members of the educated public the opportunity to contribute to the common stock of observations and experiments on subjects as diverse as natural history, agriculture, medicine and the practical arts. The market for such magazines expanded in the latter part of the eighteenth century as reading audiences grew and as the book trade became more entrepreneurial in exploiting new possibilities. In the process, special audiences began to be demarcated by magazine titles, including ladies, various religious groups and such occupational groups as medical men and those engaged in agriculture.

On the Continent, magazines were being directed at those with specifically ‘philosophical’ interests from the 1770s, but in Britain it was not until the 1790s that there seemed to be an adequate market to warrant such publications, and within a decade five had been founded.^3^ The new journals were produced by men on the fringes of the learned world of science—most notably chemist, author and inventor, William Nicholson, and newspaper proprietor and inventor, Alexander Tilloch—who saw in them a steady source of income and a means of securing their reputations. Moreover, titles such as Nicholson's *Journal of Natural Philosophy, Chemistry and the Arts* (1797–1813) and Tilloch's *Philosophical Magazine* (1798–) made clear how radically they differed from existing learned publications. Where costly learned transactions were typically slow to appear, and carried original and often extensive memoirs that had been submitted to the relevant academy, the new cheap journals appeared monthly and sought to draw together a conspectus of the state of scientific discovery, combining original contributions with extensive translations and abridgements of memoirs from across the Western world. Quite unlike scientific journals of the early twenty-first century, these early scientific journals marketed themselves as anthologies of discovery for practical men whose lack of financial and linguistic resources, and time, kept them from accessing the great range of European learned transactions. Nevertheless, these were important venues for the publication of original scientific work, especially in rapidly changing fields such as electrochemistry, and attracted original contributions by a wide range of prominent men of science, such as Humphry Davy, John Dalton and William Hyde Wollaston.^4^

These, then, were the first publications designated as ‘scientific journals’ in English, but it is not my intention here to offer an alternative origin story for *the* scientific journal, as though it were an essential and unchanging feature of modern science. On the contrary, my point is that, although the contents of these ‘scientific journals’ were subsequently indexed in the Royal Society's *Catalogue of Scientific Papers* (1867–1925), they were radically unlike modern scientific journals. Publications more recognizably like modern scientific journals did not begin to appear in Britain until the last quarter of the nineteenth century, exploiting the emergence of a new market among university-based research scientists.^5^ Moreover, the history of scientific journals has been a history of continual reinvention, as both the project of natural enquiry and the culture of communication have undergone profound change.

The constant reinvention of the scientific journal is well exemplified by the attempts of editors and publishers to re-engineer the form in the years after 1813. Several of the new philosophical journals of the 1790s had proved a modest commercial success, selling in the order of a thousand copies per month. As a result, leading publishers were soon jostling for the opportunity to purchase Tilloch's journal, which fashionable publisher John Murray considered ‘one of the most respectable and best-selling of our periodical publications’. In the event, Tilloch's overproduction of stock copies proved too discouraging, but the fashionable publishers continued to keep their eye on the growing market, and following the end of the Napoleonic Wars sought to enter it.^6^ In a few short years, four new philosophical journals were being issued by leading publishers: Robert Baldwin's *Annals of Philosophy* (1813–1826, edited by chemist Thomas Thomson), John Murray's *Journal of Science and the Arts* (1816–1830, edited by chemist William Thomas Brande), Archibald Constable's *Edinburgh Philosophical Journal* (1819–1864, edited by natural philosopher David Brewster and Edinburgh's professor of natural history, Robert Jameson) and William Blackwood's *Edinburgh Journal of Science* (1824–1832, again edited by Brewster). In each case, the publisher had responded to the initiative of an ambitious university-educated man of science as editor, and it is clear that these editors were in part motivated by the prospect of enhanced personal reputation and of improved communication between those who recognized each other as active and competent figures in the increasingly specialized sciences—those who soon after began to be referred to as the ‘working men of science’. In addition, however, both publishers and editors were motivated by the prospect of the significant income that they considered could be secured by tapping into a significantly increased market.

Why did an increased market for scientific journals seem possible in the years after 1813? In part, this reflected a general expansion and diversification of reading audiences in Britain that had been under way and accelerating since the latter part of the eighteenth century. Increasingly, entrepreneurial booksellers found among the middle and even the working classes of the growing urban centres of industrializing Britain groups of readers whose interests could be developed to make a market for new products. Periodicals were an especially effective way of exploring sectional markets, and the number of periodical titles doubled between 1800 and 1830.^7^ Moreover, there was a growing market for scientific publications more generally, including scientific books for use in schools and universities as well as encyclopaedias, scientific dialogues and naturalists’ journals for use in middle-class homes. It was in this context that publishers sought to carve out an increased market for the scientific journal, investing heavily in order to produce a quality product that would yield good returns. These were, however, experiments, and as this second generation of British scientific journals hit the market with their ambition for greater sales, they posed again some enduring questions. Who were scientific journals for? What was their proper mode of address? What kind of literature were they?

The answer that publishers and editors reached reflected two innovative distinctions. First, in their attempt to divide readers into sectional interest groups who would buy products with different contents and modes of address, they came to distinguish in a new way between what they increasingly saw as scientific and literary audiences. In place of the unitary monthly magazine, they now saw an opportunity to sell readers both scientific and literary magazines. Secondly, in their attempt to produce a scientific audience that would be large enough to render their magazines commercially viable, publishers and editors invoked an emerging notion that scientific subjects might need to be rendered ‘popular’—or, in an ugly neologism, ‘popularized’. Where the first generation of scientific journals had found a regular but small market among those interested in engaging with the technicalities of scientific subjects, the second generation hoped to expand that market by finding a more ‘popular’ mode of address that would engage a wider range of readers. These two distinctions—between the scientific and the literary and between the popular and the technical—are abiding features of modern culture, but they are far from being timeless or unchanging. On the contrary, the experiments in publishing scientific journals that took place in the years after 1813 mark a key moment in the first emergence of these important distinctions.

The notion that the new scientific magazines entailed a conscious separation of scientific and literary products was outlined in November 1824 in the ground-breaking ‘literary’ monthly, *Blackwood's Edinburgh Magazine*. An anonymous article, penned by the Unitarian civil servant William Stevenson, reviewed the ‘reciprocal influence’ between periodicals and intellectual progress in the years since the French Revolution of 1789. With reading audiences growing and diversifying, a ‘division of labour’ in periodical literature had become necessary:

Hence the contents of the Magazines became divided; and instead of a Magazine being the repository of papers on a great variety of topics, literary, technical, domestic, &c., it was found that almost every one department was sufficient to support and fill its own peculiar Magazine. Thus, we now see such a variety of these periodical publications: the mechanic, the chemist, the man who dabbles in physic, &c. &c. has his own Magazine.^8^

Stevenson—whose daughter Elizabeth later achieved renown as the novelist Mrs Gaskell—emphasized that this new specialization in periodical literature had entailed the separation of ‘scientific’ and ‘literary’ audiences and modes of address, noting that ‘those who might more strictly be called literary men—men who cultivated their judgment and taste with no ulterior object in view but the high gratification which they thus secured to themselves’ had become ‘more numerous, and required that their Magazine should no longer admit papers, useful and instructive indeed, but devoted to subjects below the level of their intellectual habits and pursuits’. The separation of scientific and literary magazines was a matter of mutual interest to those who wished to read strictly scientific journals and to ‘that literary class of readers’ who were ‘no longer content to possess Magazines only partly devoted to them’. Stevenson argued that it was the magazine in which he was writing—*Blackwood's*—that had led the way in developing the new ‘literary’ magazine, devoting itself solely to the gratification of readers of judgement and taste, and excluding scientific matter.

It would be easy to read into Stevenson's account modern notions of the distinction between the scientific and the literary that would be anachronistic. As numerous scholars have shown, the distinction between the two has been historically complex and variable. Nevertheless, it is clear that the growing association between imagination, creativity and literature that was characteristic of the Romantic movement, became for some a means of distinguishing printed products in a crowded market. This occurred as poets and natural philosophers debated the question in their writings, as publishers continued to issue ‘literature’ that was ‘scientific’ and as institutions wrestled with the proper connection of the ‘literary and philosophical’ and the ‘arts and sciences’ that often appeared in their names.^9^ It was in this highly fluid context that the relationship between the new scientific journals and their audiences had to be established. Moreover, that commercial process undoubtedly redounded on the wider cultural debate.

For publishers, the separation of the scientific magazine from the literary magazine was a strategy to exploit a growing market by diversifying products. A key question, however, was whether there would be enough readers to sustain this increased range of products. How many readers would there prove to be for scientific journals? The leading fashionable publishers were sanguine. One after another, they concluded that an able, scientifically reputable editor who was well-paid for his labours and who had ample money to spend in securing high-quality contributions from other scientific men, should be able to put together a journal that would sell several thousand copies per month and bring in a decent financial return. Moreover, they appear to have been correct since, as the number of titles increased, overall sales likewise increased. Yet, with growing competition, individual journals increasingly struggled to sell in the anticipated numbers, and publishers began to cast around for ways to increase sales. Why did the journals not sell better? The publishers concluded that they were too forbiddingly technical. For editors such as Thomson, Brande, Jameson and Brewster, it was important that their journals provide the ‘working men of science’ with opportunities to learn about the latest findings in detail and in turn to engage with them, and the new journals continued to receive articles from leading men of science. As publishers knew, however, the number of such readers was distinctly limited, and many of those who might be interested in reading about the sciences in a monthly magazine did not share their passion for the arcane details. A more ‘popular’ mode of address would widen the appeal of the new journals, making them commercially viable.

While completely familiar to modern readers, the notion of a ‘popular’ mode of address—and of ‘popular science’ in particular—was strikingly novel in the early nineteenth century. As publishers sought to expand and diversify the market for print, especially among the middle and working classes, they began to use the word ‘popular’ to demarcate products in terms of their cheapness or accessibility. The phrase ‘popular science’ in particular began to be used to demarcate certain kinds of cheaper or more accessible publications, including the scientific schoolbooks that now began to be sold in much larger numbers.^10^ It was in this context that fashionable publishers hoped to develop a large market for their new scientific journals by capitalizing on a much broader readership than might be interested in the transactions of the new specialist scientific societies that were characteristic of the period.

The situation was well characterized by a reviewer of the *Edinburgh Philosophical Journal* in the *Scotsman* in June 1824. The article claimed that ‘the multiplication of learned societies, and the rapid progress of discovery’ had ‘rendered Scientific Journals more indispensable than at any former period’. The ‘leading object’ of such journals should be, the writer claimed, to provide ‘clear and popular accounts of the discoveries made at home and abroad’, showing their value to the unlearned as well as the learned. Drawing attention to the importance of the market, the reviewer continued:

Philosophical journals ought to be addressed not exclusively to men of profound science, who are few in number, and will not be satisfied with the scraps they get in such works, but to the mass of persons whom business or curiosity interest in scientific pursuits, without having taste or time for deep researches. Such journals should be considered as the links that connect the learned with the industrious—the strainers and digesters through which the truths of philosophy must pass to fit them for assimilating with the system of active and busy life.^11^

They should ‘assume the critical and didactic tone of our leading Reviews, translating the results of abstruse researches, locked up in formulae and diagrams, into popular language, shewing us how we may be the wiser or the richer for our knowledge’.

This vision of the market for the new scientific journals was one shared by their fashionable publishers, and to some extent by their editors. From the start, however, there was a significant degree of tension in the combination of purposes. Where the newly emerging distinction between the ‘literary’ and ‘scientific’ served to locate *different products* aimed at *different audiences*, the distinction between the ‘popular’ and the ‘technical’ served to locate *different audiences* for a *single product*. While the new editors were willing to write or commission articles aimed at a broad audience, they prided themselves on securing original contributions to science, contrasting their journals in this regard with those that had gone before. As we shall see, the difficulty of combining these purposes proved too much for them in practice, especially as the market diversified further in the 1820s, with new journals that more explicitly separated ‘popular’ and ‘technical’ audiences. In the face of this development, all hope of a large market for accessible but scientifically authoritative monthly magazines seemed to be lost, and the fashionable publishers beat a hasty retreat.

## Reimagining the form and purpose of the scientific journal, 1813–1825

By the time the war with France ended in 1815, ‘scientific’ or ‘philosophical journals’ had become firmly established as a recognized feature of the British magazine trade. Of the 30 contemporary magazines listed by the *Literary Panorama* in 1807, three were journals of the arts and sciences (the *Philosophical Magazine*, the *Journal of Natural Philosophy* and the *Repertory of Arts*), two were serialized natural history publications (the *Botanical Magazine* and the *Naturalist's Miscellany*) and one was a medical magazine (the *Medical and Physical Journal*). The other specialized magazines comprised the *Agricultural Magazine*, the *Naval Chronicle* and the *Sporting Magazine*, in addition to three ladies’ and seven religious magazines.^12^ Into this market came four new scientific journals in the space of a dozen years, all published by well-established publishers and edited by university-educated men of science, and all pitching to establish themselves as market leaders in what seemed to be a potentially lucrative line. By the end of 1832, however, only one was still in existence. In this section, I briefly analyse this transitional moment in the history of the scientific journal from the predominantly commercial perspective of the publishers and editors involved. Focusing on the four new scientific journals in turn, I examine how their projectors were obliged to consider what and who the scientific journal was for, and how specialist scientific knowledge should best be handled in public form. As we shall see, questions of this sort were heavily interconnected with questions of money, as editors and contributors sought payments that would support their scientific endeavours and as publishers attempted to capitalize on their sometimes considerable investments.

### Baldwin's *Annals of Philosophy* (1813–1826)

The first of the new scientific journals was the *Annals of Philosophy, or, Magazine of Chemistry, Mineralogy, Mechanics, Natural History, Agriculture, and the Arts*, started in January 1813 by the London publisher, Robert Baldwin, and the Scottish chemist, Thomas Thomson (1773–1852). In many ways, 1813 was an unpropitious time to be launching a new journal, with the economic rigours of the prolonged war with France and Napoleon's blockade of Britain restricting access to Continental journals, but Thomson felt that the easing of the blockade presented new opportunities for scientific communication.^13^ Moreover, both he and Baldwin saw the new venture as an opportunity to exploit a market that had been shown to be remunerative.

Like the first generation of scientific journal editors, Thomson supported himself for many years by writing, editing and lecturing, but he had received extensive formal education at the University of Edinburgh in the 1790s, including as a medical student. His interests were turned towards chemistry by Joseph Black's lectures, and, with few occupational options, he had considered a career in the Kirk. Instead, he spent the next decade supporting himself by lecturing and giving practical classes on chemistry, preparing a successful *System of Chemistry* (1802) based on articles he had written for the third supplement of the *Encyclopaedia Britannica*. Then, having advised his close friend, the philosopher James Mill, to pursue his literary ambitions in London, he joined him in commencing a new periodical there in 1803.^14^ Their *Literary Journal*—published by Robert Baldwin—was innovative, offering literary intelligence on a weekly basis. This, however, was literature in the older, more capacious sense of the term. Indeed, the prospectus laid great emphasis on ‘the rapid communication of New Discoveries in the Arts and Sciences’, which it claimed had been overlooked by Britain's periodicals.^15^

The *Literary Journal* was to be divided into three sections—literature, manners and politics—and its literature section was to be subdivided into ‘Physics’, encompassing ‘Mechanical Philosophy, Chemistry, Medicine, Mathematics, and Natural History’, and ‘for want of a better term, Literature’, encompassing ‘Theology, the Philosophy of Mind, Moral Philosophy, History, Biography, Geography, Chronology, Travels, Criticism, Poetry, &c.’. Thomson was responsible for ‘Physics’. Early numbers provided introductory remarks on the sciences, but thereafter he filled several pages of the journal with a number of generally quite short articles reporting recent findings in the sciences, typically in abstracts from learned transactions and scientific journals. By the end of the first volume, however, there was growing strain in this unified notion of literature. Mill wrote:

Physics, being addressed in a great measure to scientific people, to people acquainted with the technical phraseology of the sciences connected with the laws of the material world, have been found uninteresting or unintelligible to a great body of readers: while the men of science, particularly interested in such information, were disposed to find so small a portion of the paper devoted to that subject, for which principally *they* were desirous of receiving it.

Mill and Thomson decided to stop treating ‘Physics as a separate head’ in order to make more room for reviews. Behind the scenes, Thomson withdrew from the periodical, and the small number of scientific articles published thereafter gradually diminished to nothing.^16^ But while the *Literary Journal* soon folded, Thomson and Baldwin continued to work together on various publications, including a *History of the Royal Society* (1812) designed to complement the abridgement of the *Philosophical Transactions* in 18 quarto volumes in which Baldwin had recently invested a king's ransom. This work was sufficiently remunerative that Thomson gave up his lucrative Edinburgh lectures, moving to London in the autumn of 1812.^17^

The *Annals of Philosophy*, which the pair began to issue in January 1813, was in many respects similar to the existing scientific journals, combining original contributions with abstracts, translations and reports of work published elsewhere. Indeed, contemporaries reported that the journal had been valued because it ‘presented an epitome of all the transactions of physical science in an agreeable and readable form’.^18^ The regular sections comprised ‘original or foreign’ memoirs, analyses of scientific books (chiefly society transactions), scientific intelligence, an account of the proceedings of philosophical societies and a list of patents and meteorological tables. With the Royal Society maintaining a prohibition on note-taking, Thomson was obliged to commit to memory ‘even numerical statements’, writing them down as soon as possible after leaving the meeting.^19^ For several years, numbers typically opened with biographical memoirs, often penned by Thomson, and he also composed lengthy synoptic accounts of progress in the physical sciences similar to the synopses in his *History of the Royal Society*. This task was, he reported, ‘laborious’, because his information came from ‘so many different sources & in so many different languages’, and involved reading long papers in which there might prove to be ‘nothing … to the purpose’.^20^

In the preface to his first number, Thomson stated that his main claim to distinctiveness lay in his qualities as a learned and experienced editor, his attempts to ‘procure the most valuable continental journals with as much rapidity as is consistent with the present limited and decreased state of intercourse with other countries’ and his ability to draw on his network among the leading scientific men for contributions. How far Thomson was able to fulfil his ambition of improving the originality of British scientific journals is beyond the scope of this limited study, but by the end of the first volume his authors had included ‘some of the first scientific men in Britain’, such as chemists John Dalton, Thomas Henry and Humphry Davy and naturalists Robert Jameson and Edward Daniel Clarke.^21^ Thomson relied in part on friendship in recruiting such contributors; in addition, however, Baldwin invested in securing contributions from highly regarded scientific authors through offering payments.^22^

With Baldwin's considerable investment, sales rose at least as high as 1500 copies, and John Murray described the *Annals* as ‘one of the most popular and respectable journals of the day, which goes to every part of the country’.^23^ Such a circulation would have generated a significant sum to divide between publisher, editor and contributors, and Baldwin was certainly reluctant to relinquish the property when Thomson was appointed lecturer in chemistry at the University of Glasgow in September 1817. Editorial assistants were appointed, but after various struggles Thomson was forced to conclude that the journal needed to be edited in London and in 1820 he transferred the editorship to the Quaker chemist, Richard Phillips. By this date, moreover, the number of competing journals was such as to be causing the journal difficulties.^24^ By 1826, sales had obviously sunk to such a level as to be providing an inadequate return, and Baldwin sold the copyright for £120 to the printer and editor of the *Philosophical Magazine*, Richard Taylor, who combined the two journals.^25^

### Murray and Colburn's *Journal of Science and the Arts* (1816–1830)

Robert Baldwin was not the most flamboyantly fashionable of the literary entrepreneurs of early nineteenth-century Britain, but those who followed him in publishing scientific journals were altogether more so. First came the publisher of Lord Byron and the *Quarterly Review*, John Murray. Located at the heart of the West End and prepared to offer spectacular sums to secure the best authors, Murray had long been interested in the possibility of publishing a scientific journal. He thus responded with alacrity when approached in 1816 by the chemist William Thomas Brande about the possibility of publishing a new quarterly *Journal of Science and the Arts*.^26^ Although the new journal was initiated by Brande as a private venture, it arose out of, and partially fulfilled, the Royal Institution's long-standing aim of producing a journal. Brande was Humphry Davy's successor as professor of chemistry at the institution, and the title page bore the organization's crest and the legend ‘edited at the Royal Institution’. Some reports attributed the idea to Davy himself and he certainly made contributions at first.^27^ Moreover, while the new journal's contents presented a mixture of original articles, translations, abridgements and reports broadly similar to existing journals, it continued to draw on Royal Institution personnel and maintained an obvious bias towards researches and events there. Indeed, the journal contained a higher proportion than its competitors of articles on antiquarian subjects and travel, and in 1819 its title was changed to *Journal of Science, Literature and the Arts*, although the ‘literature’ here was still very much the older sense of ‘general literature’, rather than Stevenson's sense of the literature of taste.

When it came to publishing the journal, Murray was an obvious choice for Brande, both as a leading publisher and as a near neighbour in Albermarle Street. Moreover, he offered handsome terms. Brande was to receive half the profits as well as considerable sums to pay for contributions, including those he wrote himself. This rose from £50 per number (an average of around four guineas per sheet of 16 pages) to considerably larger sums, and Brande reported that some of the contributors had ‘very capacious maws’. Murray was not only to supply payment for contributors, but was also to provide Brande with contributions from his circle. However, the publisher did require ‘important alterations’ in the plan, which Brande thought would make it less useful. In particular, the journal was to observe some restrictions in regard to reviews of scientific works, apparently so as not to interfere with Murray's *Quarterly Review*. At first the journal satisfied Murray's ambitious hopes, with its print run rising to 2000 copies in March 1819, and the publisher made a concerted effort to promote it using trade discounts. By 1822, however, sales were falling, probably because of the advent of the *Edinburgh Philosophical Journal* in what was already a crowded market.^28^ Certainly Brande was concerned by the competition, concluding that he needed to ‘work hard to make the Journal *popular*’ in order to ‘beat Brewster & his crew out of the field’.^29^ But sales continued to decline, and with the journal making a loss and Murray's business otherwise in trouble, the publisher pulled out in 1826.

Brande now engaged with another fashionable publisher, Henry Colburn, for a new series of the journal. The terms closely resembled those which seem to have operated with Murray. Brande and Colburn were to share both the copyright and the profits of the enterprise equally. The editor was to write at least three sheets (48 pages) himself and was to receive £63 to pay contributors, a sum which would rise to £84 if the circulation reached 1500 copies and £150 if it reached 2000. Clearly with the intention of increasing circulation, however, the journal was ‘to be conducted upon a more extended and popular plan than before’, and ‘instead of being chiefly confined to the abstract Sciences and principally addressed to the proficient’, its contents were to be made ‘more suitable for the general reader’.^30^ This intention was set out in a notice to readers in the last number of the old series, which detailed the alterations that would make the journal ‘more universally useful and popular’. The scientific news and miscellaneous intelligence were to be extended, and Brande promised to strip ‘the valuable facts of science of the *verbiage* and prolixity in which they are sometimes enveloped’. Despite the changes, however, the journal continued to fall short of expected sales, and in 1830 Brande was reportedly earning very little, if anything, by his labours.^31^

The end came when the managers of the Royal Institution decided to publish a journal more immediately under their own management.^32^ According to Faraday, the commencement of the replacement *Journal of the Royal Institution* (1830–1831) reflected a desire for a journal that was ‘truly scientific’ and would include ‘as much foreign science as possible’, something that had been ‘sadly neglected’. On this occasion Murray was to publish on commission, and he would ‘of course have his profits’, but the Royal Institution would use any proceeds to pay handsomely for contributions. Even then, however, the popular mode of address was important to the journal's success, and it was not long before Faraday was telling German chemist Eilhard Mitscherlich that what was needed for the journal was something ‘popular but scientific’ that was ‘not too profound & mathematical but yet clear & good & fit to be an authority’.^33^ Moreover, the new journal did not operate with the same imperative to achieve a large readership that would generate a profit for the publisher, but its financial difficulties (and Faraday's editorial reluctance) soon led to its cessation.^34^

### Constable's *Edinburgh Philosophical Journal* (1819–1864)

The most compelling manifestation of the growing conviction in this period that the market for scientific journals was potentially highly lucrative is provided by the *Edinburgh Philosophical Journal*. The first scientific journal in Scotland to attract the backing of leading literary entrepreneurs or to achieve any degree of success, it became the scene of a fierce contest between Edinburgh's leading publishers, William Blackwood and Archibald Constable. Edinburgh had been set alight in 1817 by the launch of *Blackwood's Edinburgh Magazine*, which, by radically reworking the monthly magazine format to provide a much more racy mixture of prose articles, poetry and fiction, had achieved both notoriety and commercial success. As Constable strove to find an answer to his rival's success, he began in 1818 to plan a scientific journal, offering Edinburgh's professor of natural history, Robert Jameson, and the printer and naturalist, Patrick Neill, ‘the most liberal terms’ as editors. The pair, however, declined. They did not, they felt, possess the ‘requisite knowledge of Natural Philosophy & Chemistry’, and they were in any case too busy.^35^ In fact, Jameson was already occupied providing contributions on natural history for *Blackwood's Magazine*, which, while it ultimately led the way in the development of the self-consciously literary magazine, at this early period still maintained a steady complement of articles on the sciences.^36^

Another of *Blackwood's* most active scientific contributors was David Brewster. Like Thomson, Brewster was a university-educated man of science who had considered the Kirk and, in the absence of a professorial appointment, had become financially dependent on literary work—notably editing Blackwood's *Edinburgh Encyclopaedia* (1808–1830). To such a man, a scientific journal promised both regular remuneration and authority, and he suggested such a publication to Blackwood in October 1818. The publisher tried ‘tempting’ him instead to make a much larger regular contribution to *Blackwood's Magazine* and to ‘receive a large sum for it’.^37^ After taking a half-share in that magazine in August 1818, John Murray had told Blackwood that he thought that its ‘prominent feature’ should be ‘literary and scientific news, and most of all the latter, for which your editors appear to have little estimation’, since this was ‘ten times more interesting to the public than any other class of literature at present’. He wanted ‘foreign literature, particularly German’ and ‘*news* in all departments’. Blackwood seems to have taken the comments to heart, and the following month one of his main contributors was asking Murray if he had no scientific man about him who could translate or abstract short items from German periodicals for him.^38^

Brewster, however, persisted. His plan, as he explained to Murray, was that, ‘in conjunction with several eminent men of science’ he would produce a quarterly philosophical journal that would be such as to ‘take its station beside the Quarterly and Edin^r^ Reviews’ and might even be superior to them in having distinguished Continental writers. Britain's scientific journals, he claimed, had ‘always been the *worst in Europe*’ and they were currently in decline, with Thomson intending to edit his work from Glasgow and the *Philosophical Magazine* about to perish. What was needed was a journal run by those fashionable publishers whose liberality had transformed British literature; it needed ‘an ample allowance to the Editor, and a certain sum for each sheet for contributors’.^39^

I am perfectly aware [Brewster wrote] that a Scientific Journal even if it were written by all the men of Genius in England would *never sell* unless it contained Science in a popular & intelligible form. A few profound & original articles are absolutely necessary to give a high character to a work of this kind; but it must be made a *readable* book for all classes of the community, and I think it would not be difficult to manage it in such a way as to be more instructive & even amusing to General Readers than any of the *two Great Reviews*. To do this is our great object. If we fail in it, the Journal will never exceed a moderate circulation; but if we succeed we do not despair of seeing on impression little inferior to that of any periodical work.

According to Brewster, existing journals devoted many pages to material that was at once technical and derivative, commonplace or simply ‘absolute Trash’. The new journal was to be written for a polite audience by men of acknowledged reputation, who would provide an authoritative guide to the progress of scientific knowledge.

Such readable fare could not be obtained on the cheap, and Blackwood was prepared to pay at ‘the rate of the highest remuneration of the day’: an editorial salary of £100 per quarterly number plus 10 guineas per sheet for 12 sheets of contributions.^40^ Having heard about Constable's rival plans, Brewster had arranged for Jameson to manage the ‘Natural History & Mineralogical department’ of his journal. Indeed, the ‘great and popular talents’ of Brewster and Jameson and ‘their most extensive correspondence’ were critical factors in Blackwood's decision to publish the journal. In the event, however, Brewster and Jameson switched the journal to Constable at the eleventh hour.^41^ Moreover, the pair's scientific contributions to *Blackwood's Magazine* thereafter quickly fell away, leaving the latter as a literary magazine largely bereft of scientific articles in just the way described by Stevenson in 1824.

The first number of Constable's *Edinburgh Philosophical Journal*, *Exhibiting a View of the Progress of Discovery in Natural Philosophy, Chemistry, Natural History, Practical Mechanics, Geography, Statistics, and the Fine and Useful Arts* appeared in June 1819, priced at 7s. 6d. In form and content, it closely resembled the *Quarterly Journal of Science*, with original articles, proceedings of societies and scientific intelligence. Yet, while Constable invested heavily in promoting it, sales never approached the anticipated levels. Blackwood had considered that the new journal would circulate at least 2000 copies, and ultimately perhaps 3000 or 4000, since the editors were ‘determined to make the work of that popular kind which general readers as well as men of science will be desirous of seeing’. Constable began production at 2500 copies per quarter, but by 1821 he was reducing this and, with profits non-existent, he had also to reduce his payments.^42^ As the editors sought to redeem their promises, they emphasized to readers that the journal was ‘the vehicle of Science in its most popular and useful form’, and that the editors had ‘exerted themselves to make a considerable portion of each Number accessible even to those general Readers whose acquirements and studies are not of a Scientific nature’.^43^

Despite their blandishments, Jameson and Brewster failed to deliver the journal of wide appeal they had promised. Renegotiating the editorial contract after five years, Constable now based his calculations on a more realistic print run of 1250 copies and sought to reduce editorial payments in consequence.^44^ At this point, Brewster tried to take the journal back to Blackwood, but Constable took legal action to assert his ownership of the copyright, continuing the *Edinburgh New Philosophical Journal* with Jameson as editor, while Brewster established his own *Edinburgh Journal of Science*. The two editors had long been at loggerheads, and now became editors of rival journals. It was Jameson, however, who sustained his more modest version of the *Philosophical Journal*, even through Constable's 1826 bankruptcy, until his death in 1854.

### Blackwood's *Edinburgh Journal of Science* (1824–1832)

Blackwood was only too pleased to make a deal with his rival's disaffected editor, and in June 1824 he advertised the first number of the *Edinburgh Journal of Science, Exhibiting a View of the Progress of Discovery in Natural Philosophy, Chemistry, Mineralogy, Geology, Botany, Zoology, Comparative Anatomy, Practical Mechanics, Geography, Navigation, Statistics, Antiquities, and the Fine and Useful Arts*.^45^ Brewster had extracted an annual editorial payment of £400, promising to render the new journal attractive to a wide readership by including what he denominated ‘popular science’, explaining that, while the ‘common objects of scientific inquiry’ were ‘generally far withdrawn from popular apprehension’, those topics that were not were appealing to both the ‘general reader’ and the ‘philosopher’. However, while Brewster's editorial salary was based on a promised circulation of 1250 copies, it never reached half that number.^46^ Before long, Blackwood withdrew from the journal and, in February 1832, Brewster suggested that the British Association for the Advancement of Science take over and amalgamate the four existing general journals of science (the *Philosophical Magazine*, the *Journal of the Royal Institution*, the *Edinburgh New Philosophical Journal*, and the *Edinburgh Journal of Science*). In the event, however, Brewster's journal was merged with the *Philosophical Magazine*, starting with the issue for July 1832.^47^

### Failure and further diversification

The university-trained editors and fashionable publishers of the new commercial scientific journals of the 1810s and 1820s had been convinced that the growing market for informed and current writing about the several sciences would support sales quite as high as the best of the literary magazines from which they were increasingly distinguished. To achieve such sales, they considered, it was essential to broaden the appeal of the scientific journal beyond a narrow audience of those engaged in scientific researches. A distinctively innovative popular mode of address was required, and the publishers were prepared to invest significant sums in paying scientific authors to make such contributions. In this, however, their success was very limited. In 1824, the reviewer of the *Edinburgh Philosophical Journal* in the *Scotsman* greeted Jameson's new series with enthusiasm as containing ‘articles on subjects of general interest, written in a popular manner, and some of them by men of acknowledged eminence’. In general, however, he considered scientific journals to be ‘much behind those which belong to the department of criticism and general literature’, both in their editing and in their contributors. Rather than being popular and useful, they were full of ‘original speculations’ by ‘fourth and fifth-rate men, upon subjects of ninth and tenth-rate importance, repulsively abstruse, and forbiddingly technical’, combined with commonplace and undigested extracts.^48^ This perception was reflected in sales. At the start of this period, in 1813, the *Philosophical Magazine* and *Journal of Natural Philosophy* jointly sold approximately 2500 copies. At first the total went up and by 1819 five journals were printing 8000 copies between them, but by 1832 the number had been again reduced to two and these were printing fewer copies than the 1813 total. The fashionable publishers’ experiment with journals that were popular as well as scientific had failed.

It is important to recognize, however, that in failing to fulfil their ambitions the new journals were operating in a market for print that was increasingly crowded and diverse. To begin with, several weekly journals were commenced in the years after 1813 that to some extent fulfilled the earlier ambitions of Thomson and Mill to combine the literary and the scientific. Thus, alongside its conventionally literary reviews, the *Literary Gazette* (founded 1817) provided regular reports of the proceedings of scientific societies and other scientific news and reviews, and others soon followed, most notably the *Athenaeum* (founded 1828). At the same period, while the new literary magazines tended to marginalize the sciences, they also relied on the scientific journals to provide the materials for ‘popular’ articles. In December 1821, the *London Magazine* offered the first of what was promised to be an occasional series under the title ‘Popular Retrospect of the Progress of Philosophy and Science’. The series was intended to ‘exhibit a comprehensive, bird's-eye view of all that is now doing by philosophers and men of science; to give an idea of the most recent improvements, as well as changes of retrogression, to our mere literary readers, and those who have not leisure to peruse the voluminous scientific Journals and Transactions daily publishing’.^49^ In the event, no more appeared, but the discussion of scientific topics in self-consciously literary periodicals aimed at wealthy and cultured readers continued to be a regular feature of the nineteenth-century periodical press.

Other new periodicals aimed at less socially exclusive audiences also soon began to offer ‘popular’ science separately from more technical matter. Beginning in 1822 with the *Mirror of Literature, Amusement and Instruction*, an array of new weekly miscellanies began to offer discussion of scientific subjects for as little as two pennies per issue. The *Mirror of Literature* regularly sold in excess of 10 000 copies, and its discussion of scientific subjects was increasingly packaged as ‘popular science’, culled from more specialist sources in bite-sized gobbets appealing to a non-specialist audience.^50^ These first ventures in cheap journalism prefigured an explosion of new cheap magazines in the 1830s, which further developed the notion of popular science. Weeklies such as the *Penny Magazine* of the Society for the Diffusion of Useful Knowledge and *Chambers’ Edinburgh Magazine*, both commenced in 1832, offered copious accounts of contemporary science that were at once cheaper and more accessible than those available in the scientific journals. Meanwhile, moreover, London's specialist scientific societies, beginning with the Astronomical Society and the Geological Society of London in 1827, began to take upon themselves the task of publishing regular accounts of their own proceedings separately from their more ponderous transactions. At the same time, a series of more populist scientific periodicals began to appear that appealed more effectually to poorer or less scientifically erudite readers, ranging from the *Mechanics’ Magazine* (founded 1823) to the *Magazine of Natural History* (founded 1828). In the face of this proliferating range of avowedly ‘popular’ and ‘specialist’ scientific journals, it is little wonder that the second wave of publishers and editors struggled to fulfil their ambitious attempt to combine different reading audiences in producing a unified scientific journal of general appeal.

## Conclusion

These largely failed experiments in producing widely read commercial science journals in the decade after 1813 offer insights of importance to both historians and scientists. For both, they provide additional evidence and a salutary reminder of the historical variability of scientific journals. Rather than being stable in form and function, scientific journals have been the product of contingent circumstances as editors, publishers and organizations have sought to meet a range of different ends, including such a basic end as the desire to make money. The focus here has been especially on the commercial context of several experimental journals, and one aspect of the wider historiographical significance of this study is the way in which it exposes the centrality of commercial motives in understanding processes of change in scientific communication in general and of scientific journals in particular. Just as at the present day, the judgement of publishers concerning how a financially viable readership might be secured and how profits might be maximized has loomed large in the history of scientific communication. Historians keen to address the role of communication in the making of scientific knowledge in the ways recently proposed by James Secord neglect such commercial considerations at their peril.^51^

A second key claim of this paper is that such commercial processes were implicated in two fundamental conceptual transitions in relation to the development of reading audiences for science in the modern era. As publishers sought new markets for their products, they developed key terminology to assist them—the ‘literary’, the ‘scientific’ and the ‘popular’—which has had lasting consequences for the way in which the scientific project is understood. This vocabulary, like the experimental periodicals themselves, was variable and unstable, but its development was central to the emergence of the disciplinary sciences in this period—what has sometimes been called the second scientific revolution. At the heart of that process was the separation of the disciplinary sciences from imaginative literature that is associated with what came to be termed Romanticism and the separation of specialist science from a more or less ignorant wider public considered capable only of consuming derivative ‘popular science’. What this paper has shown is that these large-scale changes in conceptualizing the nature of, and audience for, science cannot be fully understood without reference to the commercial context in which they emerged.

While the modern world has inherited this key vocabulary and the distinctions they imply, that has not occurred without further change. Indeed, as editors and publishers continued to explore the market for scientific journals through the course of the nineteenth century, their categories for organizing and apprehending readers continued to develop. At mid century, for instance, new monthly ‘literary’ reviews, such as the *Contemporary* and *Fortnightly*, provided important platforms for leading men of science to contribute to intellectual debate, while leading publisher, Alexander Macmillan, pitched his new scientific weekly, *Nature*, at a wide audience, knowing that only by making it to some degree ‘popular’ could he make it financially viable. Only with the emergence of the academic research scientist towards the end of the nineteenth century did publications and their associated audience categories begin to settle into something more recognizably modern. Even now, however, notions of what it is to be ‘scientific’, ‘literary’ and ‘popular’ are deeply entangled in the practices and interests of commercial publishing. Thus, a third aim of this paper has been to focus attention on the need for science historians and literary scholars to explore further the history of this complex conceptual entanglement.

That historical project naturally connects to the current debate concerning the future of the scientific journal. The account given here of the journal's historical variability serves to enliven the imagination regarding possibilities for the future. There is no timeless answer to the questions of who should write and read about science, and of the form and purposes of such writing. The notion that the only significant audience for original scientific research consists of highly trained specialists based largely in academic institutions and reading scientific journals is a relatively recent one, and it is one that is again being questioned. Indeed, the sentiment of the 1820s—that ‘Philosophical journals ought to be addressed not exclusively to men of profound science … but to the mass of persons whom business or curiosity interest in scientific pursuits’—seems to chime with the ethos of public engagement that has prevailed in Britain since the publication of the House of Lords report *Science and Society* in 2000.^52^ Ours is a world in which a well-educated public both can be and often expects to be engaged in high-level debate about the purposes and priorities of the sciences in ways that are not trivial for the future of scientific research. Just as in the early nineteenth century, such readers cannot necessarily be expected to read arcane and expensive journals, and in such circumstances one might anticipate a new role for publications, including journals, that address multiple audiences. The science journals designed for a wide audience in early nineteenth-century Britain may have failed to find their market, but their history may thus yet serve to prompt scientists of the early twenty-first century to reimagine the scientific journal once again.

## Notes

1 *The Future of Scholarly Scientific Communication*, 2015, https://royalsociety.org/~/media/events/2015/04/FSSC1/FSSC-Report.pdf (accessed 19 January 2016).

2 See, for example, James A. Secord, ‘Science, technology and mathematics’, in *The Cambridge history of the book in Britain*, vol. 4 *1830–1914* (ed. David McKitterick), pp. 451–459 (Cambridge University Press, 2009); Alex Csiszar, ‘Broken pieces of fact: the scientific periodical and the politics of search in nineteenth-century France and Britain’, PhD thesis, Harvard University (2010); Jonathan R. Topham, ‘Anthologizing the book of nature: the circulation of knowledge and the origins of the scientific journal in late Georgian Britain’, in *The circulation of knowledge between Britain, India and China: the Early-Modern world to the twentieth century* (ed. Bernard Lightman, Gordon McOuat and Larry Stewart), pp. 119–152 (Brill, Leiden and Boston, 2013); Iain Watts, ‘“We Want No Authors”: William Nicholson and the contested role of the scientific journal in Britain, 1797–1813’, *Brit. J. Hist. Sci.* **47**, 397–419 (2014); Melinda Baldwin, *Making* Nature*: the history of a scientific journal* (University of Chicago Press, 2015).

3 Topham, *op. cit*. (note 2); Thomas Broman, ‘Periodical literature’, in *Books and the sciences in history* (ed. Marina Frasca-Spada and Nicholas Jardine), pp. 225–238 (Cambridge University Press, 2000); James E. McClellan III, ‘The scientific press in transition: Rozier's journal and the scientific societies in the 1770s’, *Ann. Sci.* **36**, 425–449 (1979); Maurice Crosland, *In the shadow of Lavoisier: the ‘Annales de Chimie’ and the establishment of a new science* (British Society for the History of Science, [n.p.], 1994).

4 Watts, *op. cit.* (note 2); S. Lilley, ‘“Nicholson's Journal” (1797–1813)’, *Ann. Sci.* **6**, 78–101 (1948–1950).

5 Secord, *op. cit.* (note 2).

6 Thomas Constable, *Archibald Constable and his literary correspondents: a memorial*, 3 vols (Edmonston and Douglas, Edinburgh, 1893), vol. 1, pp. 352–354, 358; Murray to Constable, 30 November 1807, National Library of Scotland (hereinafter NLS), John Murray Papers, Out-Letters, MS.41908, p. 152.

7 Geoffrey Cantor *et al.*, *Science in the nineteenth-century periodical: reading the magazine of* *nature* (Cambridge University Press, 2004), pp. 7–10.

8 [William Stevenson], ‘On the reciprocal influence of the periodical publications, and the intellectual progress of this country’, *Blackwood's Edinburgh Magazine* **16**, 518–528 (1824), at p. 524.

9 Raymond Williams, *Keywords: a vocabulary of culture and society* (Fontana Press, London, 1988), pp. 183–188; Ralph O'Connor, *The earth on show: fossils and the poetics of popular science, 1802–1856* (University of Chicago Press, 2007), esp. introduction; Sharon Ruston, *Creating Romanticism: case studies in the literature, science and medicine of the 1790s* (Palgrave Macmillan, Basingstoke, 2013), esp. introduction; Jon Klancher, *Transfiguring the arts and sciences: knowledge and cultural institutions in the Romantic age* (Cambridge University Press, 2013).

10 Jonathan R. Topham, ‘Publishing “popular science” in early nineteenth-century Britain’, in *Science in the marketplace: nineteenth-century sites and experiences* (ed. Aileen Fyfe and Bernard Lightman), pp. 135–168 (University of Chicago Press, 2007); Topham, ‘Rethinking the history of science popularization/popular science’, in *Popularizing science and technology in the European periphery, 1800–2000* (ed. Faidra Papanelopoulou, Agusti Nieto-Galan and Enrique Perdiguero), pp. 1–10 (Ashgate, Aldershot, 2009). For an invaluable discussion of the use of the term ‘technical’ in relation to science, see Theodore M. Porter, ‘How science became technical’, *Isis* **100**, 292–309 (2009); the use of the term in relation to these early journals invites further consideration of its relation to the emerging use of ‘popular’.

11 ‘Edinburgh Philosophical Journal’, *Scotsman*, 16 June 1824, p. 8.

12 *Literary Panorama* **2** (1807), col. 65.

13 Jonathan R. Topham, ‘Science, print, and crossing borders: importing French science books into Britain, 1789–1815’, in *Geographies of nineteenth-century science* (ed. David N. Livingstone and Charles Withers), pp. 311–344 (University of Chicago Press, 2011).

14 Jack Morrell, ‘Thomson, Thomas (1773–1852)’, in *Oxford dictionary of national biography* (Oxford University Press, 2004; online edn, 2006 http://www.oxforddnb.com/view/article/27325, accessed 10 August 2016); [Robert Dundas Thomson], ‘Biographical notice of the late Thomas Thomson’, *Glasgow Med. J.* **5**, 69–80, 121–153 (1857), at p. 126. This biography is clearly that attributed to Thomson's nephew in Alexander Bain, ‘The [early] life of James Mill’, *Mind* **1**, 97–116, 509–531 (1876), **2**, 519–551 (1877), at vol. 1, pp. 509n–510n. It also quotes from numerous manuscripts described by Bain as then being owned by Thomson's family; some of these manuscripts are quoted in Alexander Bain, *James Mill: a biography* (Longmans, Green & Co., London, 1882). Thomson had earlier published a brief obituary: R. D. Thomson, ‘Memoir of the late Dr Thomas Thomson’, *Edinburgh New Philosophical Journal* **54**, 86–98 (1852–1853).

15 [James Mill and Thomas Thomson], *Prospectus of a new weekly publication, entitled ‘The Literary Journal’* (C. Baldwin, London, [1802]). A copy of the prospectus is in the John Johnson Collection, Bodleian Library, Oxford.

16 [James Mill], ‘To our readers’, *Literary Journal* **1**, cols 841–842 (1803), at col. 841; J. B. Morrell, ‘Thomas Thomson: professor of chemistry and university reformer’, *Brit. J. Hist. Sci.* **4**, 245–265 (1969), at p. 247n.

17 Thomson, *op. cit.* (note 14), p. 123; Walter Crum, ‘Sketch of the life and labours of Dr Thomas Thomson, F.R.S. president of the Philosophical Society’, *Proceedings of the Glasgow Philosophical Society* **3**, 250–264 (1855), at p. 253. The exact date of Thomson's move is uncertain. He was still described as a chemical lecturer in Edinburgh in his nomination to the fellowship of the Royal Society on 10 January 1811 (Royal Society, EC/1811/02), but signed the dedication to his Swedish travels from London on 24 April 1813.

18 Thomson, *op. cit.* (note 14), pp. 138–139.

19 Crum, *op. cit.* (note 17), p. 256.

20 Thomson to Macvey Napier, 9 November 1815, British Library, Add.MS.34611, f. 310.

21 [Thomas Thomson], ‘Preface’, *Ann. Phil.* **1**, [1]–4 (1813), at p. 4; [Thomson], ‘Advertisement’, *Ann. Phil.* **1**, [iii]–iv (1813), at p. iv.

22 See, for example, Thomson to John Dalton, 13 August 1818, in Henry E. Roscoe and Arthur Harden, *A new view of the origin of Dalton's atomic theory: a contribution to chemical history, together with letters and documents concerning the life and labours of John Dalton, now for the first time published* (Macmillan & Co., London, 1896), p. 171; and Thomson to Hans Christian Oersted, 27 January 1819, in M. C. Harding, *Correspondence de H. C. Oersted*, 2 vols (Aschehoung, Copenhagen, 1920), vol. 2, p. 557.

23 William Blackwood to Cadell and Davies, [after 9 February 1819], NLS, MS.30073, ff. 40–41; Samuel Smiles, *A publisher and his friends: memoir and correspondence of the late John Murray, with an account of the origin and progress of the house, 1768–1843*, 2nd edn, 2 vols (John Murray, London, 1891), vol. 1, p. 240.

24 Thomson, *op. cit.* (note 14), p. 142; Roscoe and Harden, *op. cit*. (note 22), p. 171; Thomas Thomson and Richard Phillips, ‘Advertisement’, *Ann. Phil.* **16** [1]–2 (1820).

25 Baldwin to Taylor, 22 and 24 July 1824, St Bride Printing Library, London, Taylor & Francis archive, II.1 Inventories; W. H. Brock and A. J. Meadows, *The lamp of learning: Taylor & Francis and the development of science publishing*, 2nd edn (Taylor and Francis, London, 1998), pp. 95–96.

26 Brande to Murray, 25 January 1820, NLS, John Murray Papers, ACC.12604/1142.

27 Topham, *op. cit*. (note 2), p. 143; Morris Berman, *Social change and scientific organization: the Royal Institution, 1799–1844* (Heinemann Educational Books, London, 1978), pp. 141–143; Frank Sulloway, ed., *Archives of the Royal Institution of Great Britain in facsimile: minutes of managers’ meetings, 1799–1900*, 15 vols in 7 (Scolar Press in association with the Royal Institution of Great Britain, Menston, 1971–1976), vol. 5, p. 111, vol. 6, pp. 78–79, 106, 127, 132, vol. 7, p. 295; Thomas Thomson to Robert Jameson, 16 February 1816, Edinburgh University Library, Gen.1999/1/151.

28 Brande to Murray, 25 January 1820 and 8 April 1826, NLS, John Murray Papers, Out-Letters, ACC.12604/1142 and Booksellers Book, 1816–1843, NLS MS.42883, pp. 1–10, 120–125, and 151–153. Murray explained the rationale behind such discounting of journals in Murray to Constable, 14 November 1809, NLS, ‘Correspondence between John Murray, London, and Archibald Constable and A. Constable & Co., Edinburgh’, MS.23233, p. 139.

29 Brande to Ure, December 1821 and 18 February 1822, Royal Institution Archives, Box File IX, Item 122E (quoted in Berman, *op. cit*. (note 27), p. 142).

30 Sulloway, *op. cit.* (note 27), vol. 7, pp. 297–299.

31 [William Thomas Brande], ‘To our readers and correspondents’, *Quarterly Journal of Science, Literature, and the Arts* **22** (1827), p. [ix]; Sulloway, *op. cit.* (note 27), vol. 7, p. 296.

32 [William Thomas Brande], ‘To Our Readers and Correspondents’, *Quarterly Journal of Science, Literature, and Art* **n.s. 7** (1830), p. [viii]. See also Sulloway, *op. cit.* (note 27), vol. 7, pp. 289–306 and 308–309.

33 Faraday to Eilhard Mitscherlich, 4 August 1830, in *The correspondence of Michael Faraday* (ed. Frank A. J. L. James), 6 vols (Institution of Electrical Engineers, London, 1991), at vol. 1, p. 524.

34 Sulloway, *op. cit*. (note 27), vol. 7, pp. 379–380; Minutes of the Journal Committee, 23 September 1831 and 16 February 1832, Royal Institution Archives, Item 102 (quoted in Berman, *op. cit*. (note 27), p. 144).

35 Blackwood to Murray, 3 November 1818, and Brewster to Murray, 13 November 1818, NLS, John Murray Archive, In-Letters, ACC.12604/1119 and 1146.

36 Alan Lang Strout, *A bibliography of articles in* Blackwood's Magazine*, volumes I through XVIII, 1817–1822* (Library, Texas Technological College, Lubbock, TX, 1959); Blackwood to Jameson, 11 June 1819, NLS, MS.30304, p. 74; Philip Flynn, ‘Beginning *Blackwood's*: the right mix of *dulce* and *ùtile*’, *Victorian Periodicals Review* **89**, 136–157 **(**2006); Flynn, ‘Beginning *Blackwood's*: the first hundred numbers (April 1817–May 1825)’, http://www.english.udel.edu/Profiles/flynn_blackwood.htm (accessed November 2007); Jonathan R. Topham, ‘*Blackwood's Edinburgh Magazine*: introduction’, in *Science in the nineteenth-century periodical: an electronic index*, v. 1.0 (hriOnline Publications, Sheffield, 2005), http://www.sciper.org (accessed 8 April 2016); William Christie, ‘*Blackwood's Edinburgh Magazine* in the scientific culture of early nineteenth-century Edinburgh’, in *Romanticism and Blackwood's* *Magazine: ‘an unprecedented phenomenon’* (ed. Robert Morison and Daniel S. Roberts), pp. 125–136 (Palgrave Macmillan, Basingstoke, 2013).

37 Blackwood to Murray, 23 October 1818, and Brewster to Murray, 23 October 1818, NLS, John Murray Archive, ACC.12604/1146; A. D. Morrison-Low and J. R. R. Christie, eds, *‘Martyr of science’:* *Sir David Brewster, 1781–1868* (Royal Scottish Museum, Edinburgh, 1984), especially the chapter by W. H. Brock.

38 Murray to Blackwood, September 1818, in Smiles, *op. cit.* (note 23), vol. 1, pp. 480–481; John Gibson Lockhart to Murray, 23 October [1818], NLS, John Murray Archive, MS.4003, ff. 133–134.

39 Brewster to Murray, 23 October 1818, NLS ACC.12604/1146.

40 Blackwood to Murray, 24 October 1818 and 13 November 1818, and Brewster to Murray, 13 November 1818, NLS, John Murray Papers, ACC.12604/1119 and 1146; Blackwood to [Cadell and Davies], [after 9 February 1819], NLS, Blackwood Papers, Out-Letters, MS.30301, f. 245; Constable & Co. to Brewster, 12 March 1821, NLS, Constable Papers, MS.791, p. 260. See also W. H. Brock, `Brewster as a scientific journalist', in Morrison-Low and Christie, *op. cit.* (note 37), pp. 36–42.

41 Brewster to Murray, 13 November 1818, and Blackwood to Murray, 3 November 1818, NLS, John Murray Papers, ACC.12604/1146 and 1119; Blackwood to Cadell and Davies (copy), [after 9 February 1819], NLS, Blackwood Papers, Out-Letters, MS.30073, ff. 40–41; [Brewster and Jameson] to Blackwood, 10 March 1819, NLS, Blackwood Papers, In-Letters, MS.30001, ff. 143–145.

42 Blackwood to Cadell and Davies (copy), [after 9 February 1819], NLS, Blackwood Papers, Out-Letters, MS.30073, ff. 40–41; Archibald Constable & Co. to Brewster, 4 October 1822, 16 October 1822, 16 January 1824 and 5 February 1824, NLS, Constable Papers, Out-Letters, MS.791, pp. 629 and 642, and MS.792, ff. 119 and 125.

43 [David Brewster and Robert Jameson], [*Advertisement for the first seven volumes of the Edinburgh Philosophical Journal*] ([Archibald Constable & Co., Edinburgh], 1822), p. [i]; a copy of the advertisement is in the John Johnson Collection, Bodleian Library. See also Constable & Co. to Brewster, 11 June 1822 and 16 October 1822, NLS, Constable Papers, Out-Letters, MS.791, pp. 563 and 642.

44 Archibald Constable & Co. to Brewster, 16 January 1824, NLS, Constable Papers, Out-Letters, MS.792, f. 119.

45 Margaret Oliphant, *Annals of a publishing house: William Blackwood and his sons, their magazine, and friends*, 2 vols (William Blackwood and Sons, Edinburgh and London, 1897), vol. 2, p. 9; *Morning Chronicle*, 25 June 1824, p. 2c.

46 [David Brewster], ‘Contributions to popular science’, *Edinburgh J. Sci.* **1** (1824), p. 37; Blackwood to Brewster (copy), 19 October 1826, NLS, Blackwood Papers, Out-Letters, MS.30302, pp. 83–87.

47 Brewster to William Vernon Harcourt, 1 February 1832, in Jack Morrell and Arnold Thackeray, eds, *Gentlemen of science: early correspondence of the British Association for the Advancement of Science* (Royal Historical Society, London, 1984), pp. 126–127; Brock and Meadows, *op. cit.* (note 25), pp. 95–96.

48 *Scotsman*, *op. cit.* (note 11).

49 ‘R’, ‘Popular retrospect of the progress of philosophy and science’, *London Mag.* **4**, 674–678 (1821), at p. 674.

50 Jonathan R. Topham, ‘The *Mirror of Literature, Amusement and Instruction *and cheap miscellanies in early nineteenth-century Britain’, in *Science in the nineteenth-century periodical: reading the magazine of* *nature* (ed. Geoffrey Cantor *et al.*), pp. 37–66 (Cambridge University Press, 2004); Topham, ‘Thomas Byerley, John Limbird and the production of cheap periodicals in Regency Britain’, *Book History ***8**, 75–106 (2005).

51 James A. Secord, ‘Knowledge in transit’, *Isis* **95**, 654–672 (2004).

52 See, for instance, *Science and society*, 3rd report of the Select Committee on Science and Technology, Session 1999–2000 (House of Lords, London, 2000) and James Wilsdon and Rebecca Willis, *See-through science: why public engagement needs to move upstream* (Demos, London, 2004). For a note of caution, see also Charles Thorpe and Jane Gregory, ‘Producing the post-Fordist public: the political economy of public engagement with science’, *Science as Culture* **19**, 273–301 (2010).

